# Decreased Gastric Motility in Type II Diabetic Patients

**DOI:** 10.1155/2014/894087

**Published:** 2014-07-23

**Authors:** Yi-Chun Chiu, Ming-Chun Kuo, Christopher K. Rayner, Jung-Fu Chen, Keng-Liang Wu, Yeh-Pin Chou, Wei-Chen Tai, Ming-Luen Hu

**Affiliations:** ^1^Division of Hepatogastroenterology, Department of Internal Medicine, Kaohsiung Chang Gung Memorial Hospital, Chang Gung University, College of Medicine, Kaohsiung, Taiwan; ^2^Division of Endocrinology & Metabolism, Department of Internal Medicine, Kaohsiung Chang Gung Memorial Hospital, Chang Gung University, College of Medicine, Kaohsiung, Taiwan; ^3^University of Adelaide, Discipline of Medicine, Royal Adelaide Hospital, Adelaide, SA 5005, Australia

## Abstract

*Background*. To differentiate gastric motility and sensation between type II diabetic patients and controls and explore different expressions of gastric motility peptides. *Methods*. Eleven type II diabetic patients and health volunteers of similar age and body mass index were invited. All underwent transabdominal ultrasound for gastric motility and visual analogue scales. Blood samples were taken for glucose and plasma peptides (ghrelin, motilin, and glucacon-like peptides-1) by ELISA method. *Results*. Gastric emptying was significantly slower in diabetic patients than controls (T50: 46.3 (28.0–52.3) min versus 20.8 (9.6–22.8) min, *P* ≤ 0.05) and less antral contractions in type II diabetic patients were observed (*P* = 0.02). Fundus dimensions did not differ. There were a trend for less changes in gastrointestinal sensations in type II diabetic patients especially abdomen fullness, hunger, and abdominal discomfort. Although the serum peptides between the two groups were similar a trend for less serum GLP-1in type II diabetic patients was observed (*P* = 0.098). *Conclusion*. Type II diabetic patients have delayed gastric emptying and less antral contractions than controls. The observation that there were lower serum GLP-1 in type II diabetic patients could offer a clue to suggest that delayed gastric emptying in diabetic patients is not mainly influenced by GLP-1.

## 1. Introduction

Gastrointestinal tract disorders are common in diabetic patients [1, 2]. More than 75% of patients visiting diabetes mellitus clinics reported significant gastrointestinal symptoms [1] such as dysphasia, early satiety, reflux, abdominal pain, nausea, vomiting, constipation, and diarrhea. The symptoms may be severe enough to substantially affect quality of life, induce poor sugar control, and progress with duration of diabetes mellitus. The pathogenesis of the gastrointestinal abnormalities is multifactorial complex in nature which may involve autonomic neuropathy, motor dysfunction, glycemic control, psychological factors, and so forth and is not well understood [3]. In diabetic patients with gastrointestinal symptoms, 68% were found to have delayed gastric emptying [4] that influences the quality of life and sugar control in these patients. Owing to the fact that the pathogenesis is still poorly understood, it is rational that the effective medical treatment for these patients with diabetic dyspepsia is yet unavailable. Gastric motility is regulated by gastrointestinal motility hormones such as cholecystokinin, gastric inhibitor peptide, motilin, and ghrelin. Previous publications reported that motilin [5, 6] and ghrelin [7, 8] stimulated gastric motility and glucagon-like peptide-1 (GLP-1) inhibited gastric motility [9, 10]. We hypothesized that diabetic patients had lower motilin and ghrelin or higher GLP-1 and hence inhibited gastric motility and induced gastrointestinal symptoms. Therefore, we conducted this study to compare gastric motility and sensation between type II diabetic patients and normal controls and explore the roles of different gastric motility peptides in this motility effect.

## 2. Methods

Eleven patients (2 female) with long-standing (>5 years) type II diabetes mellitus (DM) and 11 healthy controls (2 female) of similar age (58 ± 2 years; 51 ± 5 years) andbody mass index (25.0 ± 2.2 kg/m^2^; 23.7 ± 2.5) were invited to participatein current study from August 2009 to July 2010. The basic demographic data were listed in Table 1. All patients gave written, informed consent, and the protocol was approved by both the institutional Review Board and the Research Ethics Committee of Kaohsiung Chang Gung Memorial Hospital, Taiwan (IRB 96-1583B). Patients were excluded if they had a historyof ketosis or dysautonomic or gastrointestinal symptoms determined by interview or from the review of medical records. All patients with type II diabetes mellitus in current study were prescribed with oral hypoglycemic agents instead of insulin therapy. Subjects were also excluded if they had previous gastrointestinal disease or abdominal surgery with the exception of uncomplicated appendectomy, herniorrhaphy, or gynecologicsurgery. All patients must discontinue medications including anticholinergics, calcium channel blockers, 3-adrenergicantagonists, or hormones 48 hours before the study except oral hypoglycemic agents. They underwent physical examination, laboratory blood tests (including white-cell and red-cell counts, measurement of blood sugar during fasting, and liver-function tests), abdominal ultrasonography, and upper gastrointestinal endoscopy to rule out any structural cause for the symptoms.

Each subject was studied on afternoons. Following a fast of 8 h for solids and liquids, the patients consumed 500 mL of chicken and corn soup (United Kanboo, Taipei, Taiwan), containing 118.6 kcal (2.6 g protein, 2.6 g fat, 21.2 g carbohydrate). The soup was boiled and subsequently cooled to 37°C and was consumed over 5 min (*t* = −5–0 min). All patients underwent transabdominal ultrasound to record antral area, fundic area, and diameterandantral contractions in regular time by using an Aloka SSD-2000 CL Ultrasound Machine (Aloka, Tokyo, Japan) with a 3.5-MHz annular array probe. The frequency of antral contractions was defined as the number of contractions during a 25-minute interval, recorded per five minutes, and the antral contractions were defined as one time while the maximal contraction-induced reduction of the antral area (difference between relaxed and contracted area) as a fraction of the relaxed area (Δ*A*/*A*) > 50% as our previous study [11]. All subjects were invited to denote their symptoms 10 min before test meal ingestion and 10 min after test meal ingestion. A questionnaire with visual analogue scales (VAS) for the symptoms of pain, nausea, abdominal discomfort, bloating, and abdominal fullness was administered every ten minutes till 90 min. Grading was made on a 100 mm unmarked line between “no symptom” at one end and “excruciating symptoms” at the other. Blood samples were taken at *T* = −10, 30, 60, and 90 minutes for measurement of blood glucose and plasma peptide levels. Blood glucose concentrations were immediately determined using a portable blood glucose meter (MediSense Companion 2 meter; MediSense Inc., Waltham, MA, USA). The accuracy of this method has been confirmed using the hexokinase technique. Venous blood samples were collected into ice-chilled EDTA-treated tubes containing 400 KIU aprotinin/mL blood. Plasma was separated and samples stored at −70°C for subsequent analysis of GLP-1, ghrelin and motilin concentrations. Plasma ghrelin, motilin, and GLP-1 concentrations were measured by ELISA (Ghrelin was measured by a commercial ELISA kit (Phoenix Pharmaceuticals Inc., Burlingame, CA); intra- and interassay coefficients of variation (CV) were less than 5% and less than 9%, respectively; motilin and GLP-1 were also measured by a commercial ELISA kit.

## 3. Statistical Analysis

The curves for antral area, fundic area, diameter, antral contractions, and gastrointestinal sensation scores were compared by using repeated measures ANOVA. Linear regression analysis was used to examine relationships between gastrointestinal sensations and gastric motility (antral area, fundic area, diameter, and frequency of antral contraction). Results were shown as means ± standard deviation. *P* values <0.05 were considered significant. Mean values and standard deviations (SD) of each variable were calculated if not specifically noted otherwise. Statistical analysis was performed with Student's *t*-test for paired comparison. The tests are two-tailed, and *P* < 0.05 is considered significant.

## 4. Results

All subjects tolerated the study well.

### 4.1. Antral Area and Gastric Emptying

**Figure 1:**
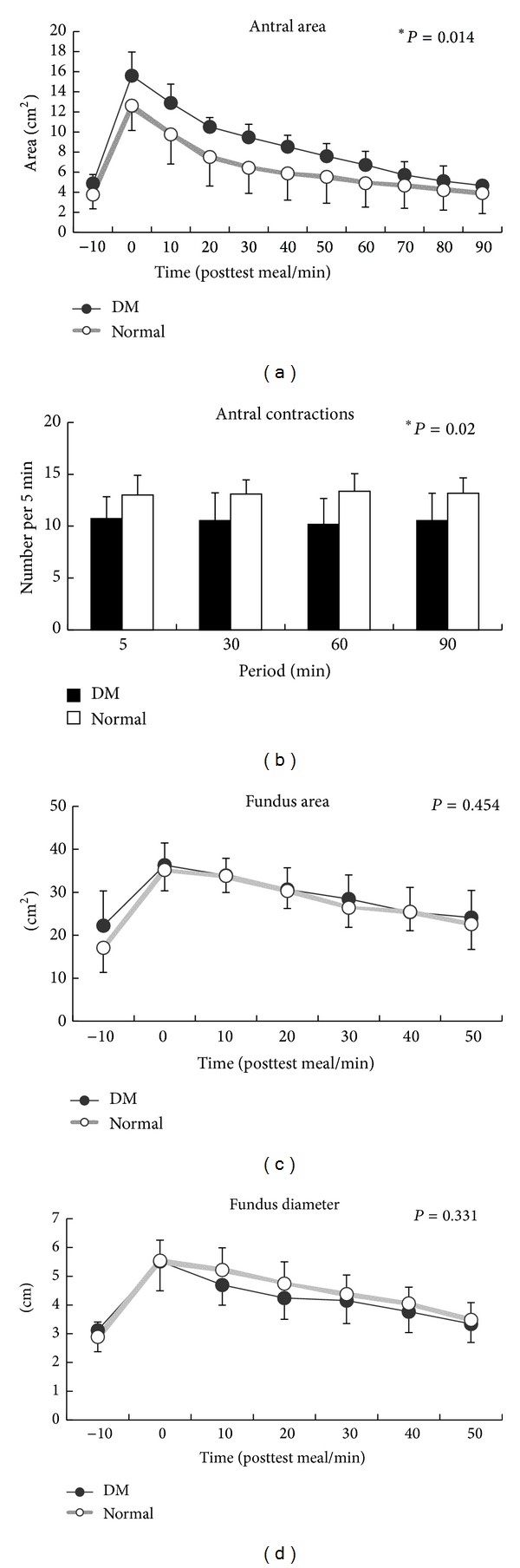
(a) There was a statistical significance trend for smaller antral area in the normal controls between the two groups; *P* = 0.014. (b) There was a significant trend for less antral contractions in type II diabetic patients during 90 minutes after the test meal (*P* = 0.02). (c) Fundus dimensions did not differ between normal controls and type II diabetic patients (fundic area: *P* = 0.454; fundic diameter: *P* = 0.331).

Antral area was larger in type II diabetic patients than normal controls especially after test meal (antal area 15.6 ± 2.3 cm^2^ versus 12.6 ± 2.4 cm^2^). Gastric emptying was more significant in normal controls than diabetic patients (T50: 20.8 (9.6–22.8) min versus 46.3 (28.0–52.3) min, *P* = 0.03). There was a statistical significance trend for smaller antral area in the normal controls between the two groups, *P* = 0.014 (Figure 1(a)).

### 4.2. Antral Contractions

There was a significant trend for less antral contractions in type II diabetic patients during 90 minutes after the test meal (*P* = 0.02) (Figure 1(b)).

### 4.3. Fundic Area and Diameter

Fundus dimensions did not differ between normal controls and type II diabetic patients (fundic area: *P* = 0.454; fundic diameter: *P* = 0.331) (Figures 1(c) and 1(d)).

### 4.4. Gastrointestinal Sensations

**Figure 2:**
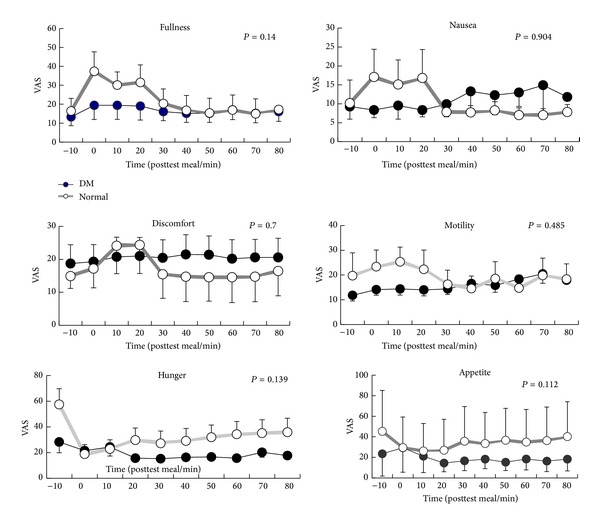
A trend for less significant changes in gastrointestinal sensation in type II diabetic patients especially abdomen fullness, hunger, and appetite in current study (abdomen fullness (*P* = 0.14), hunger (*P* = 0.139), appetite (*P* = 0.112), nausea (*P* = 0.904), discomfort (*P* = 0.7), and motility (*P* = 0.485)).

Soup ingestion was associated with increased abdominal fullness and bloating and decreased hunger and appetite scores only in control group but not in type II diabetic patients.  Figure 2 demonstrated a trend for less significant changes in gastrointestinal sensations in type II diabetic patients especially abdomen fullness, hunger, and appetite in current study.

### 4.5. Gastric Motility Peptides

**Figure 3:**
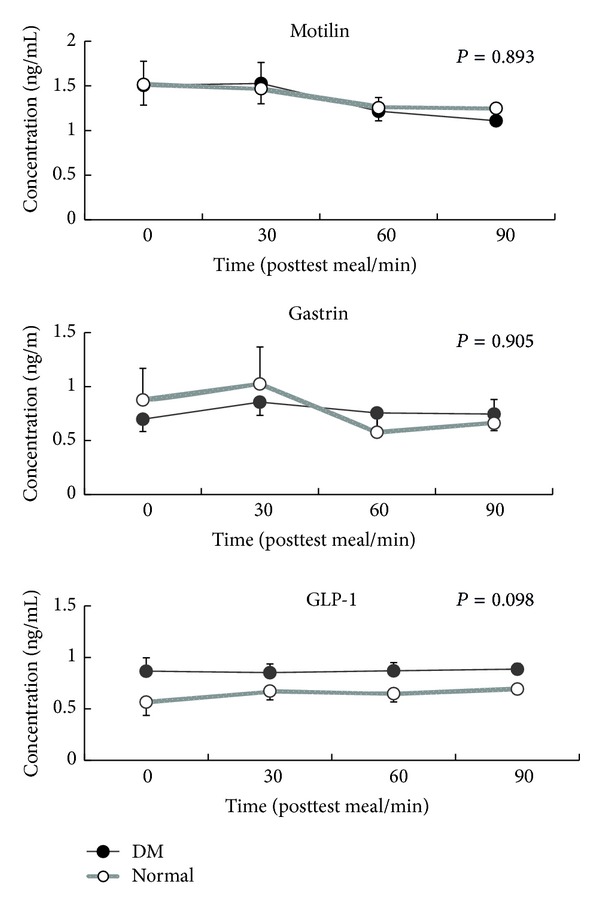
There were no differences in serum peptides (motilin, ghrelin, and GLP-1) between two groups but a trend for less serum GLP-1 in patients with type II diabetes mellitus was observed (*P* = 0.098).

There were no differences in serum peptide (motilin, ghrelin, and GLP-1) between the two groups but a trend for less serum GLP-1 in patients with type II diabetes mellitus was observed (*P* = 0.098) (Figure 3).

## 5. Discussion

Faraj and colleagues reported as high as 68% of the diabetic patients with gastrointestinal symptoms were associated with delayed gastric emptying [4]. In current study, we observed that type II diabetic patients were found to have larger gastric antral area on sonography. Type II diabetic patients had delayed gastric emptying and less gastric contraction and experienced less postprandial sensation than normal volunteers probably owing to gastric atony and gastric paralysis (gastroparesis) in diabetic patients.

As we had shown that less gastric contraction in diabetic patients were correlated with less gastric emptying, there were reports indicating that diabetic patients had postprandial antral hypomotility, decreased temporal organization of antral pressure waves, and increased small intestinal motor activity. All these gastric disorders occurred in long-term diabetic patients and were associated with peripheral neuropathy. Prolonged poor sugar control induced sympathetic nerve dysfunction. Samsom and colleagues observed a lower fasting fundal tone and a decrease in volume change of the gastric fundus after a nutrient drink in patients with autonomic neuropathy due to type I diabetes mellitus but the fundus dimensions between type II diabetic patients with normal controls were not different in current study. They used barostat to measure fundic volume and tone but we used ultrasound to detect fundic dimensions. Kumar and colleagues then emphasized more significant impairment in gastric fundic volume and the accommodation response to a test meal in diabetic patients [12] but we still did not observe the difference in our study. The plausible explanation could be the low nutrient test meal we used in current study. Low volume test meal would not remain in the fundus long enough to be detected by abdominal ultrasound. Perhaps such drawback could be overcome if higher nutrient test meals were used. Other than this, computer tomography might be a better imaging study to overcome the difference.

Type II diabetic patients experienced less gastrointestinal sensation after a test meal than normal controls in spite of significant delaying gastric emptying and decreasing gastric contraction. Loo and colleagues found out that approximately 50% of these patients with gastric motor disorders might be asymptomatic [13].

It is still unclear about the actual roles of different motility expression in diabetic patients. Motilin had been reported to regulate the interdigestive migrating contractions (IMC), the fasted motor pattern in the gastrointestinal tract [14], and ghrelin stimulated GI motility. Ghrelin induced premature phase III contractions of IMC in the human stomach [7] and intravenous ghrelin injection could accelerate gastric emptying and improve meal-related symptoms. All these reports suggested that ghrelin could be a potential prokinetic agent. More studies are mandatory to clarify the physiological role of plasma ghrelin and motilin in these digestive physiological events and stimulation of gastric emptying. However, we observed no different motilin and ghrelin between diabetic patients and normal controls, only a trend of less serum GLP-1 expression in type II diabetic patients in current study. This could not well explain the pathology of delayed gastric emptying in our diabetic patients. This was consistent with the findings of Pala and colleagues who reported that GLP-1 levels were significantly lower in subjects with impaired glucose tolerance and type 2 diabetes mellitus compared to those with normal glucose tolerance [15]. Shirra and Delgado-Aros and colleagues observed that GLP-1 significantly inhibited gastric emptying and vagal function [16, 17]. Rosenstock and colleagues even used GLP-1 agonists to treat diabetic patients [18, 19]. There was limitation in our study, small patient number that could cause why there was no difference in motility peptides in current study.

In conclusion, Type II diabetic patients have delayed gastric emptying and less antral contractions than normal controls and may be associated to less postprandial sensation. The observation that less serum GLP-1 in type II diabetic patients could offer a clue to understand that delayed gastric emptying in diabetic patients is not mainly regulated by GLP-1.

## Figures and Tables

**Table 1:** The basic characteristics between type II DM and normal control.

	Type II DM (11)	Normal controls (11)	*P *
Age (years)	58 ± 2	51 ± 5	NS
Gender (female/male)	2/11	2/11	NS
BMI (kg/m^2^)	23.7 ± 2.5	25.0 ± 2.2	NS
Duration of DM (years)	11.1 ± 5.6	0	0.000∗
Fasting glucose	126.0 ± 32.1	82.0 ± 4.9	0.012∗
HbA1C	9.5 ± 1.3	5.5 ± 0.1	0.000∗

*Significant statistic difference.
